# Vanishing Bile Duct Syndrome in the Presence of Hodgkin Lymphoma

**DOI:** 10.7759/cureus.26842

**Published:** 2022-07-14

**Authors:** Pramod Gaudel, Paige Brown, Ken Byrd

**Affiliations:** 1 Department of Hematology and Oncology, University of Kansas Medical Center, Kansas City, USA; 2 Internal Medicine, University of Kansas School of Medicine, Kansas City, USA

**Keywords:** hodgkin lymphoma, supraclavicular lymph node, classic hodgkin lymphoma, clinical complete response, vanishing bile duct

## Abstract

Vanishing bile duct syndrome (VBDS) is an acquired condition characterized by the destruction and loss of intrahepatic bile ducts resulting in cholestasis. VBDS has been described in various conditions including neoplastic and immunologic disorders, infections, hepatic ischemia, and drug toxicity. The diagnosis is confirmed by liver biopsy revealing the loss of interlobular bile ducts in greater than 50% of portal tracts. Prognosis is variable and often unpredictable but appears to be influenced by the etiology of bile duct destruction and overall patient health. VBDS has been described as a rare paraneoplastic process in patients with Hodgkin lymphoma. This case describes a 26-year-old female who presented with a neck mass, jaundice, and pruritus. Initial workup revealed direct hyperbilirubinemia, transaminitis, elevated alkaline phosphatase, and elevated international normalized ratio. She went on to receive a diagnosis of stage II classical Hodgkin lymphoma, nodular sclerosing subtype, and biopsy-proven VBDS. Over the course of chemotherapy, complete metabolic resolution of Hodgkin lymphoma and complete normalization of bilirubin were achieved. She was given gemcitabine and cyclophosphamide as a liver sparing regimen initially with some improvement in liver function tests and a reduction in lymph node volumes. She received six cycles of adriamycin/bleomycin/vinblastine/dacarbazine (ABVD) with complete remission attained after four cycles by positron emission tomography/computed tomography criteria. This report illustrates asafe chemotherapy regimen in the presence of marked liver dysfunction. Workup for VBDS including liver biopsy should be pursued in Hodgkin lymphoma patients with evidence of cholestasis in the absence of extrahepatic bile duct damage or other known etiology of liver injury.

## Introduction

Vanishing bile duct syndrome (VBDS) encompasses a group of acquired conditions that are characterized by progressive destruction and loss of intrahepatic bile ducts and the development of cholestasis. VBDS has been described in several neoplastic disorders, immunologic disorders, allograft rejection, infections, hepatic ischemia, and drug toxicity. VBDS is a well-established but rare paraneoplastic syndrome seen in Hodgkin lymphoma. Clinical manifestations of VBDS vary based on etiology but frequently include jaundice, pruritus, fatigue, anorexia, abdominal pain, and weight loss. Laboratory abnormalities including elevated alkaline phosphatase, elevated gamma-glutamyl-transferase (GGT), predominantly direct hyperbilirubinemia, and transaminitis support the diagnosis. A liver biopsy is necessary to confirm the loss of intrahepatic bile ducts. The resulting cholestatic liver damage from VBDS presents a challenge in selecting a chemotherapy regimen for patients with Hodgkin lymphoma. This report illustrates a case of VBDS in the presence of Hodgkin lymphoma safely treated by one cycle of gemcitabine plus cyclophosphamide as a liver-sparing regimen followed by a total of six cycles of adriamycin/bleomycin/vinblastine/dacarbazine (ABVD) with complete remission.

## Case presentation

A 26-year-old female graduate student with a medical history significant only for mild scoliosis presented to the hospital with the chief complaint of right neck mass associated with neck pain radiating to the right arm with numbness, tingling, and visible veins on the right upper arm and right chest. She also reported dark urine, pale-colored stools, jaundice, and pruritus for approximately two weeks. She denied B symptoms including fever, night sweats, and unintended weight loss. Her history was unremarkable for human immunodeficiency virus or any other immunosuppressive conditions. She noticed the right neck mass eight to nine months prior to the presentation. It was thought to be reactive lymphadenopathy at that time. Unfortunately, due to a lack of insurance and access to medical care, she did not receive further evaluation. Family history was unremarkable for lymphoproliferative disease, myeloproliferative disease, or other malignancies. She denied any history of tobacco use, illicit substance use, current alcohol use, and use of any medications within two weeks. Over the course, she had tried various over-the-counter medications with no improvement in her symptoms.

On initial physical examination, the patient was found to be markedly jaundiced with bilateral icteric sclera and a firm mass in the right supraclavicular area which was tender to palpation. There were prominent veins on the proximal right upper extremity and across the upper chest anteriorly. The remainder of the examination was unremarkable. Laboratory results were significant for total bilirubin of 22.0 mg/dL, direct bilirubin of 14.5 mg/dL, alanine aminotransferase (ALT) and aspartate aminotransferase (AST) of 91 U/L and 108 U/L, respectively, alkaline phosphatase of 662 U/L, international normalized ratio (INR) of 1.3, and lactate dehydrogenase of 257 U/L. The remaining laboratory results including complete blood count, basic metabolic panel, serum electrophoresis, immunoglobulin levels, hepatitis panel, coronavirus disease 2019, Epstein-Barr virus, HIV, cancer antigen 19-9 (CA-19-9), alpha-fetoprotein, and urinalysis were within normal limits.

**Figure 1:**
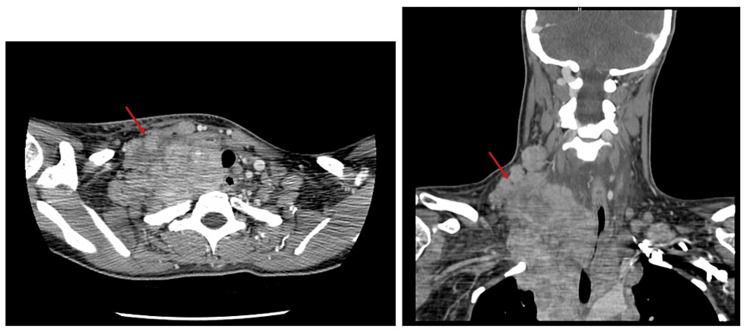
Computed tomography of the neck with contrast: ill-defined soft tissue mass (red arrows) diffusely involving the upper mediastinum, right supraclavicular fossa, and right inferolateral neck.

**Figure 2:**
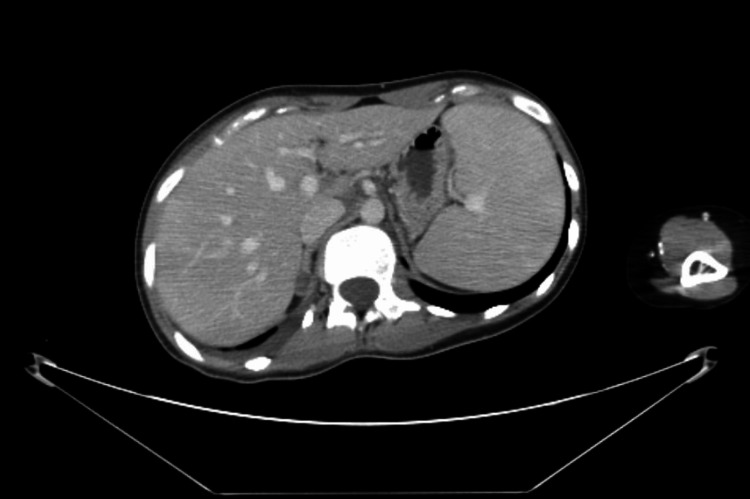
Computed tomography of the abdomen/pelvis with contrast: mild hepatomegaly, moderate splenomegaly, and a nondistended gallbladder.

**Figure 3:**
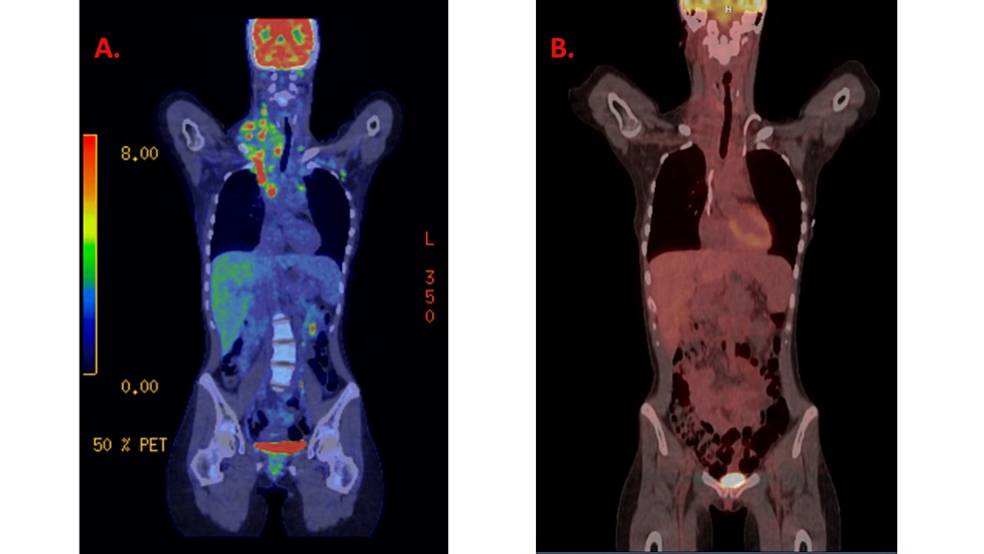
(A) Positron emission tomography scan at the time of presentation. (B) Positron emission tomography scan after two cycles of chemotherapy.

A computed tomography (CT) of the neck and chest showed a large heterogeneous right neck mass measuring 9.9 × 6.6 cm (Figure 1) and extending into the mediastinum and right lung apex with bilateral cervical and thoracic lymphadenopathy with occlusion of the right brachiocephalic and jugular veins and moderate compression of the left brachiocephalic vein. CT of the abdomen and pelvis showed moderate splenomegaly and mild hepatomegaly with a completely nondistended gallbladder (Figure 2). Magnetic resonance cholangiopancreatography (MRCP) was unremarkable with no evidence of extrahepatic biliary dilation. Positron emission tomography (PET) scan showed extensive hypermetabolic supradiaphragmatic lymphadenopathy, including a large nodal mass within the right neck and superior mediastinum with associated mass effect and infiltration of the brachial plexus, left internal mammary, sternum, and probable left apical pulmonary involvement (Figure 3, Panel A). Ultrasound-guided core needle biopsy of the right neck mass was obtained which revealed classic Hodgkin lymphoma, nodular sclerosis type. Immunohistochemical staining showed tumor cells positive for CD30 and CD15 and negative for CD3, CD5 Pan-Mel (human melanoma black-45, melanoma-associated antigen recognized by T cells), pan-cytokeratin, CD10, anaplastic lymphoma kinase, and CD45. No morphologic or immunophenotypic evidence of lymphoma was found on a bone marrow biopsy. There were no clonal chromosome abnormalities by conventional cytogenetics. A liver biopsy was obtained due to the physical examination and laboratory evidence of cholestasis. It showed prominent cholestasis and Kupffer cell hyperplasia with focal perivenular hepatocyte dropout and 54% loss of bile ducts (duct loss in seven of 13 portal tracts) without direct hepatic involvement by lymphoma which was supportive of paraneoplastic VBDS in the setting of Hodgkin lymphoma.

During the patient’s initial hospitalization, therapy options were limited due to the degree of hyperbilirubinemia. She was started on intravenous (IV) dexamethasone 20 mg IV × four days. She received one cycle of inpatient chemotherapy consisting of IV gemcitabine 800 mg/m^2^ and IV dexamethasone 20 mg (day one) and IV gemcitabine 800 mg/m^2^ and IV cyclophosphamide 750 mg/m^2^ (day eight). She was also initiated on ursodeoxycholic acid (UDCA) due to reported benefits in various etiologies of cholestasis. Total bilirubin fluctuated over the course of hospitalization and ranged from 20.9 to 25.0 mg/dL. AST ranged from 76.0 to 268 U/L. ALT ranged from 90.0 to 316 U/L. AST and ALT generally increased for four to five days following chemotherapy then declined. Alkaline phosphatase fluctuated but trended down from 662 U/L on admission to 341 U/L upon discharge. Her symptoms significantly improved and she was discharged with an outpatient chemotherapy plan at another nearby medical center (due to insurance status).

The patient then completed six cycles of ABVD. Rituximab was added to the first two cycles due to case reports of success against autoimmune bile duct injury [1]. Vinblastine was reduced by 50% during cycle one. Rituximab was dosed at 375 mg/m^2^, adriamycin at 25 mg/m^2^, bleomycin at 10 units/m^2^, vinblastine at 3 mg/m^2^ for cycle one and 6 mg/m^2^ for the remaining cycles, and dacarbazine at 375 mg/m^2^. She was continued on UDCA through cycle three. Total bilirubin trended down over cycle one from 26.0 mg/dL to 10.0 mg/dL. Jaundice and right supraclavicular swelling resolved by the end of cycle two. A PET scan was performed following two cycles of ABVD. The dominant mass significantly decreased in overall size from 6.1 × 4.8 cm to 4.8 × 2.2 cm in the greatest axial dimension. There was a significant decrease in metabolic activity and essentially complete interval resolution of the mediastinal lymphadenopathy with no abnormal fluorodeoxyglucose (FDG) accumulation. No new abnormal mass lesions or FDG accumulation were demonstrated (Deauville score 2, Figure 3, Panel B).

**Figure 4:**
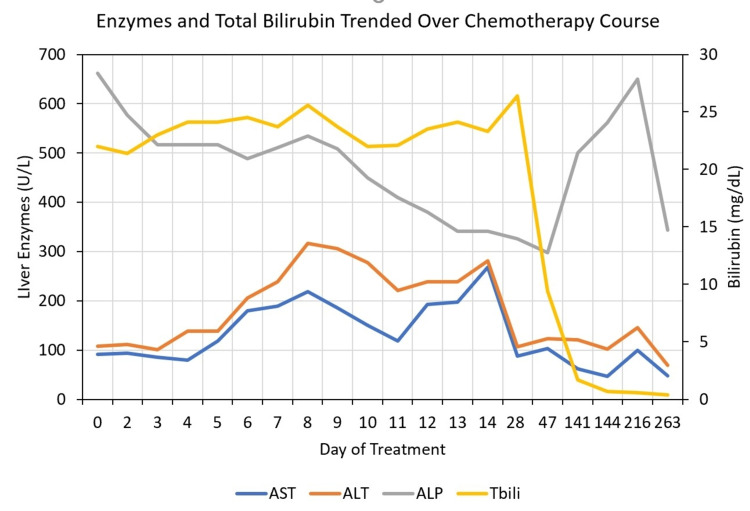
Trend of liver enzymes and bilirubin levels over the treatment course. AST: aspartate transaminase; ALT: alanine transaminase; ALP: alkaline phosphatase; Tbili: total bilirubin

**Figure 5:**
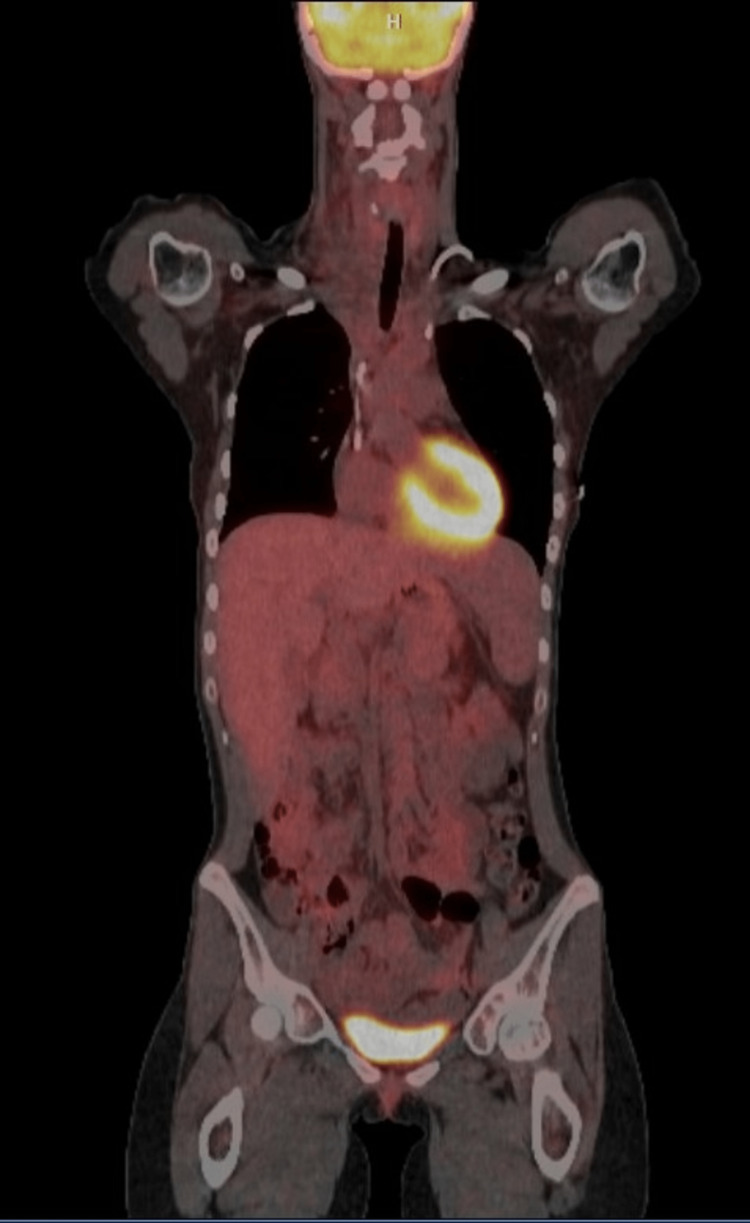
Positron emission tomography scan after four cycles of chemotherapy, showing complete metabolic response, Deauville score 2.

Complete normalization of total bilirubin was achieved by the end of cycle three, although AST and ALT remained elevated (Figure 4). UDCA was discontinued at this time. The patient tolerated chemotherapy well up to this point, experiencing only mild peripheral neuropathy of bilateral ring fingers. She was admitted after the fourth cycle for neutropenic fever and mucositis at which time she was started on cefepime and metronidazole. Symptoms resolved and she was discharged. PET scan performed following four cycles showed FDG avidity equal to blood compatible with complete metabolic response, Deauville score 2 (Figure 5). She was then planned to receive two additional cycles of ABVD to complete a total of six cycles to avoid radiation to the chest, given her young age.

The patient went on to receive two additional cycles of ABVD without complication and remained in complete remission by PET/CT criteria. Post-treatment CT of the neck, chest, and abdomen/pelvis demonstrated ill-defined left and right supraclavicular soft tissue fullness corresponding to known malignancy but no new evidence of lymphadenopathy. Bilirubin remained within normal limits, though AST and ALT remained mildly elevated at 146 U/L and 100 U/L, respectively.

## Discussion

The mechanism of VBDS has been described in the literature to be due to variety of hepatic insults resulting in progressive destruction and disappearance of intrahepatic bile ducts and ultimately leading to cholestasis [2]. VBDS has a variety of etiologies represented in Table 1 [3].

**Table 1:** Causes of vanishing bile duct syndrome.

Category	Examples
Medication	Ampicillin, amoxicillin, flucloxacillin, erythromycin, tetracycline, doxycycline, cotrimoxazole
Infection	Human immunodeficiency virus
Immune-mediated	Primary biliary cholangitis, primary sclerosing cholangitis, autoimmune hepatitis, graft-versus-host disease, sarcoidosis
Malignancy	Lymphoma (B-cell, T-cell rich B-cell, Hodgkin, non-Hodgkin, anaplastic large cell)
Other	Histiocytosis, ischemia, intrahepatic chemotherapy

The association between VBDS and Hodgkin lymphoma was first explained in 1993 in three patients with intrahepatic cholestasis and ductopenia by Hubscher et al. [4]. The proposed pathophysiology to explain Hodgkin lymphoma-related VBDS is an immune-mediated insult of the biliary epithelium from the autoantibodies produced by the lymphoma or T-cell-mediated toxicity and direct damage to the bile ducts by cytokines [2,5]. Due to paucity in the understanding of the pathophysiology of VBDS, there is not a diagnostic marker or a test, and the syndrome remains a diagnosis of exclusion. Prognosis is variable and depends on the degree of biliary epithelial cell apoptosis and capacity for biliary regeneration. VBDS could be progressive leading to biliary cirrhosis and liver failure. Even when liver failure does not occur, VBDS in Hodgkin’s may predict poor prognosis.

Liver infiltration in Hodgkin lymphoma ranges from 5% to 8% but the range has been as high as 30-70% [6]. Although VBDS is a rare phenomenon, liver failure from VBDS has a high mortality in an otherwise curable malignancy. Usually, VBDS treatment is the intervention of the underlying cause. But treatment of Hodgkin lymphoma becomes challenging when associated with VBDS. However, the need of curative chemotherapy that may be limited by the extent of liver dysfunction and progressive cholestasis must be balanced. Multiple factors can have impact on patient’s prognosis including prior liver disease, alcohol abuse, disease stage, performance status, degree of hepatic dysfunction, and other comorbidities. It has been previously reported in literature that about one-third of patients with VBDS in Hodgkin lymphoma could have a reversible disease course with appropriate treatment [7]. The most commonly used regimen in Hodgkin lymphoma is ABVD; however, there is no specific guideline or consensus whether the dose of chemotherapy should be reduced [8]. Anthracyclines and vinca alkaloids are metabolized in the liver and are relatively contraindicated in patients with severe liver dysfunction. Other drugs such as gemcitabine and carboplatin which are renally excreted could safely be used in patients with liver dysfunction. Hence, initial treatment with gemcitabine, carboplatin, cyclophosphamide, mechlorethamine, or steroids is recommended until liver functions improve. After liver functions are stabilized, ABVD can be used. This patient was initiated on dexamethasone 20 mg IV for four days upon diagnosis. Initial chemotherapy consisted of IV gemcitabine 800 mg/m^2^ (day one) and IV gemcitabine 800 mg/m^2^ plus IV cyclophosphamide 750 mg/m^2^ (day eight) as a liver-sparing regimen. About two weeks later, the patient received two cycles of R-ABVD followed by four cycles of ABVD. Vinblastine was reduced by 50% during cycle one. Rituximab was dosed at 375 mg/m^2^, adriamycin at 25 mg/m^2^, bleomycin at 10 units/m^2^, vinblastine at 3 mg/m^2^ for cycle one and 6 mg/m^2^ for the remaining cycles, and dacarbazine at 375 mg/m^2^. The use of UDCA has been shown to help improve liver functions in these patients [8]. Typically, the improvement in liver functions lags the control of malignancy. Patients can have persistent mild liver function abnormalities for some time despite being in complete remission.

## Conclusions

Care should be taken to perform a thorough workup for VBDS in Hodgkin lymphoma patients with evidence of cholestasis without an alternative explanation. This case highlights an important concept that timely initiation of an appropriate chemotherapy regimen despite the presence of significant liver dysfunction can be lifesaving in critically ill patients with VBDS in the presence of Hodgkin lymphoma.
